# Advances in Antibody-Based Therapeutics for Cerebral Ischemia

**DOI:** 10.3390/pharmaceutics15010145

**Published:** 2022-12-31

**Authors:** Jui-Ming Sun, Ting-Lin Yen, Jing-Shiun Jan, Pharaoh Fellow Mwale, Ruei-Dun Teng, Rajeev Taliyan, Cheng-Ta Hsieh, Chih-Hao Yang

**Affiliations:** 1 Section of Neurosurgery, Department of Surgery, Ditmanson Medical Foundation, Chia-Yi Christian Hospital, Chia-Yi City 600, Taiwan; 2 Department of Biotechnology, Asia University, Taichung City 41354, Taiwan; 3 Department of Pharmacology, School of Medicine, College of Medicine, Taipei Medical University, No. 250, Wu Hsing St., Taipei 110, Taiwan; 4 Department of Medical Research, Cathay General Hospital, Taipei 22174, Taiwan; 5 Neuropsychopharmacology Division, Department of Pharmacy, Birla Institute of Technology and Science, Pilani 333031, India; 6 Division of Neurosurgery, Department of Surgery, Sijhih Cathay General Hospital, New Taipei City 22174, Taiwan; 7 School of Medicine, National Tsing Hua University, Hsinchu 300044, Taiwan; 8 Department of Medicine, School of Medicine, Fu Jen Catholic University, New Taipei City 24205, Taiwan

**Keywords:** antibody, cerebral ischemia, ischemic, hemorrhagic, blood brain barrier

## Abstract

Cerebral ischemia is an acute disorder characterized by an abrupt reduction in blood flow that results in immediate deprivation of both glucose and oxygen. The main types of cerebral ischemia are ischemic and hemorrhagic stroke. When a stroke occurs, several signaling pathways are activated, comprising necrosis, apoptosis, and autophagy as well as glial activation and white matter injury, which leads to neuronal cell death. Current treatments for strokes include challenging mechanical thrombectomy or tissue plasminogen activator, which increase the danger of cerebral bleeding, brain edema, and cerebral damage, limiting their usage in clinical settings. Monoclonal antibody therapy has proven to be effective and safe in the treatment of a variety of neurological disorders. In contrast, the evidence for stroke therapy is minimal. Recently, Clone MTS510 antibody targeting toll-like receptor-4 (TLR4) protein, ASC06-IgG1 antibody targeting acid sensing ion channel-1a (ASIC1a) protein, Anti-GluN1 antibodies targeting N-methyl-D-aspartate (NMDA) receptor associated calcium influx, GSK249320 antibody targeting myelin-associated glycoprotein (MAG), anti-High Mobility Group Box-1 antibody targeting high mobility group box-1 (HMGB1) are currently under clinical trials for cerebral ischemia treatment. In this article, we review the current antibody-based pharmaceuticals for neurological diseases, the use of antibody drugs in stroke, strategies to improve the efficacy of antibody therapeutics in cerebral ischemia, and the recent advancement of antibody drugs in clinical practice. Overall, we highlight the need of enhancing blood–brain barrier (BBB) penetration for the improvement of antibody-based therapeutics in the brain, which could greatly enhance the antibody medications for cerebral ischemia in clinical practice.

## 1. Introduction

According to the World Health Organization, stroke is generally referred to as the result of decreased or interrupted blood supply to the brain due to a blocked or ruptured artery, with neurological deficits persistent for over 24 h [1,2]. This undersupply results in a lack of nutrients or oxygen, causing brain cells to die. Globally, stroke is one of the leading causes of death, with the number of people affected being higher in low- and middle-income countries than in high-income countries [3]. According to the American Health Association’s 2020 Update, 1 in 19 deaths in the United States is attributable to stroke; the World Health Organization calls stroke the epidemic of the 21st century [4,5,6]. The risk factors for cerebral ischemia are similar to those for coronary and other vascular diseases. Therefore, effective therapeutic strategies targeting the major modifiable factors such as hyperlipidemia and diabetes would be helpful in reducing stroke risk. In addition, lifestyle risk factors can also be addressed [7]. Combined prevention strategies have been shown to be effective in reducing deaths from stroke even in some low-income countries [8,9]. Strokes are mainly classified into two types: ischemic strokes and hemorrhagic strokes, with transient ischemic attacks (TIAs) being a subtype of ischemic strokes and more than 80% of strokes being ischemic [10,11,12,13].

In an ischemic stroke (IS), there is a rapid decrease in blood supply to the brain, immediately depriving the brain of oxygen and glucose. The lack of blood supply leads to the death of neurons and glial cells, with subsequent loss of brain function. The incidence of a stroke triggers several pathological events, including the activation of glutamate receptors, leading to the release of glutamate and the influx of calcium ions, which activate nitric oxide, caspases, and proteases. This activation causes inflammation, free radical formation, and protein damage leading to neuronal cell death [14]. Other highlights of stroke include blood–brain barrier (BBB) damage, oxidative stress, cytokine-mediated toxicity, excitotoxicity, and loss of neuronal function [15]. Since stroke is the leading cause of death and neurological disability worldwide, new and effective therapies are critical [16,17]. Cerebral ischemic stroke is divided into thrombotic and embolic strokes, where thrombotic ischemic stroke is caused by blood vessel narrowing and embolic ischemic stroke is associated with blood vessel blockage that reduces blood flow to the brain [18]. However, the simultaneous activation of multiple signaling pathways makes the treatment of stroke challenging. Stroke treatment must focus on resolving blood clots, clearing blockages, and simultaneously treating the associated complications in the brain. Currently, the only pharmacologic intervention for treating ischemic stroke in the brain is tissue plasminogen activator (tPA), which dissolves the blood clot, improves blood flow, and is delivered directly to the brain. Surgical intervention is another option [16,19,20]. Another important treatment for cerebral ischemic stroke is recanalization, a technique to remove blood clots from the brain after ischemic stroke. After making a small incision in the groin, a physician threads a thin tube (catheter) through the blood vessels to the clot. A tiny device at the tip of the catheter grabs the clot and removes it, restoring blood flow to the brain. Recanalization is indicated in patients with persistent occlusion after intravenous thrombolysis. The occluded environment promotes inflammatory signals and contributes to tissue damage [21], warranting combination therapy as recommended in the STAIR guidelines [22,23]. Antibody-based therapies have been tested against various inflammatory molecules. Therefore, antibodies, when used in synergy, could help reduce inflammation and thus promote brain protection. The synthetic drug has been approved by the United States Food and Drug Administration (US-FDA) for IS. However, tPA must be administered less than 4.5 h after the onset of symptoms, which limits its effectiveness; the outcome can also be fatal if it is administered outside 4.5 h [24,25]. In addition to the narrow therapeutic window of tPA, more than 50% of patients receiving tPA acutely after stroke have significant long-term disability [25]. tPA, if not administered at the correct time and dose, can cause intracerebral hemorrhage, which can eventually lead to death. Intracerebral hemorrhage is referred as bleeding into the parenchyma of the brain, which may extend into the ventricles and, in rare cases, into the subarachnoid space. This type of hemorrhage accounts for 10% to 15% of all strokes and is associated with the highest mortality rate, with only more than 30% of affected patients surviving the first year [26]. Cerebral hemorrhagic strokes are defined as ischemic regions with a variable amount of blood cells leaking from damaged vessels, either due to increased permeability of the vessel walls or post-ischemic vessel rupture. Affected brain regions exhibit continuous blood leakage, resulting in hypoxia (due to decreased blood supply), increased intracranial pressure (which in turn negatively affects cerebral blood flow), and persistent irritation. These phenomena are considered even more severe than ischemic stroke. Subarachnoid hemorrhage is a less common subtype of stroke that results from the rapid rupture of a basal cerebral aneurysm [27]. This type of damage attracts white blood cells that further damage brain tissue. Treatment strategies are based on lowering intracranial pressure, controlling cerebral blood flow, removing malformations, and catheterization to drain excess cerebrospinal fluid to lower intracranial pressure [28,29]. Another treatment method is surgical cutting or wrapping to stop blood flow at the base of the ruptured vessel to maintain blood supply to the brain [30,31]. Surgical treatments are the only emergency treatment options available for hemorrhagic strokes, whereas for ischemic strokes, tPA therapy is widely used in addition to surgical interventions.

## 2. Antibody-Based Therapeutics

**Figure 1:**
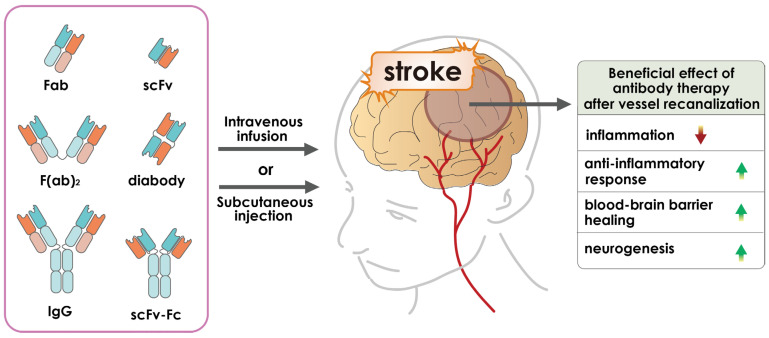
Schematic representative diagram demonstrating intravenous or subcutaneous injection of different forms of antibody-based medications to a stroke patient. The development of stroke triggers several mechanisms, including activation of glutamate receptors, which leads to the release of glutamate and influx of calcium ions that activate nitric oxide, caspases, and proteases. This activation leads to inflammation, producing free radicals, and protein damage, resulting in neuronal cell death. Other important aspects of stroke include blood–brain barrier (BBB) damage, oxidative stress, cytokine-mediated toxicity, excitotoxicity, and loss of neuronal function [14,15].

Because of the difficulties associated with tPA and the complexity of surgical procedures, it is necessary to consider other safe and effective therapeutic strategies for strokes. Therefore, new treatments are needed to improve outcomes in patients outside the 4.5-h therapeutic window of tPA [32]. Recently, accumulating evidence has indicated that targeting an endogenous molecule or signaling cascade with monoclonal antibodies would reduce the severity of damage or completely heal the damaged brain tissue (Figure 1). Antibody drugs for the treatment of stroke have attracted considerable attention in the scientific community.

After stroke, numerous signal transduction pathways are altered. Targeting certain harmful signaling pathways may delay brain damage and even increase the window of opportunity for revascularization therapy. In addition, interaction of antibodies with cytotoxic molecules and their receptors could rescue cell viability or delay cell death. Studies of immunotherapy for stroke are currently underway, including active immunization by inoculation with peptides and passive immunization by direct injection of antibodies into animals. Additionally, animal models are currently being used to research the effectiveness of targeting antibodies. For stroke treatment, a variety of molecules have been chosen as targets, and several antibodies have been created. These molecules can be accessed by antibodies since they are primarily found on or in the extracellular space of cells. A typical animal model of ischemic stroke is middle cerebral artery occlusion (MCAO). Clinical trials using numerous antibodies have failed due to subpar patient results, despite the fact that the majority of antibodies have been successful in reducing brain damage in animal models of stroke. However, despite significant advancements in brain shuttle techniques, it is still difficult for antibodies to pass the blood–brain barrier (BBB) in order to treat stroke. Bispecific transferrin receptor (TfR) antibodies, for example, have been shown to be effective in enhancing BBB exposure of several large compounds with limited brain permeability [33,34,35]. However, further improvements are needed to make this strategy a more reliable technology. Until then, cerebral ischemia medication discovery and development will continue to focus on optimizing the biophysical and binding qualities for optimal brain exposure [36]. More critically, additional clinical trials utilizing pertinent animal species and illness models are required in order to increase our understanding of the impact of antibody alteration on brain-targeted uptake and efficacy [37,38]. In this review article, we examine recent developments in the field of antibody medications for neurological disorders and which of these could potentially become an effective antibody drug for stroke treatment. Meanwhile, we also discuss the advanced methods that could increase the efficacy of antibody drugs in stroke, and the progress of antibody drugs in clinical practice.

## 3. Recent Antibody-Based Drugs for Neurological Disorders

**Figure 2:**
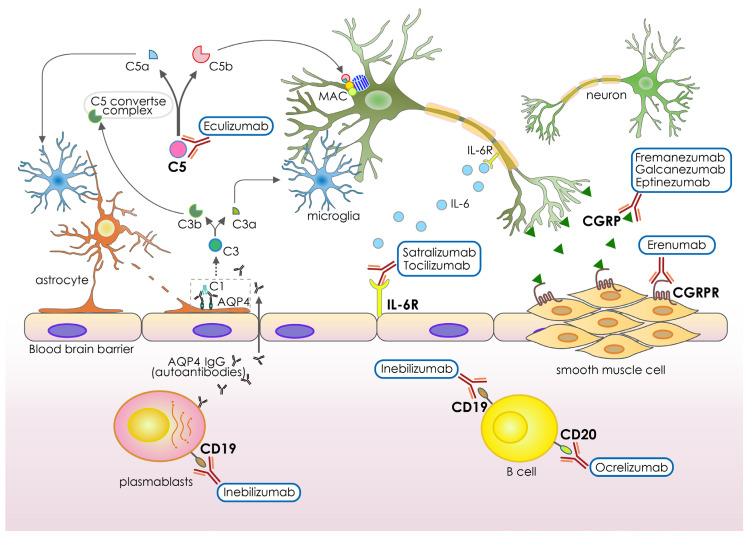
Schematic representative of recently developed antibody-based drug in different neurological diseases. Antibody-based drugs exert their therapeutic effects through a variety of mechanisms. Some antibodies block the function of the membrane receptors. Others target specific complement proteins for cell depletion. Finally, antibody-based drugs can target specific effector molecules from interacting with their ligands. Immunogens include IL-6L/R, interleukin 6 ligand/receptor; CGRPL/R: calcitonin gene-related peptide ligand/receptor; CD19/20: cluster of differentiation 19/20; C5: complement factor 5 protein.

Neurological diseases are the main cause of mortality and disability globally [39,40]. The adaptive immune system and the interactions with cells and structures in the central nervous system frequently play a role in the pathophysiology of injury and disease processes [41,42]. Because of this, there is much interest in creating neuroprotective antibody-based therapies that focus on neuroinflammatory and other signaling pathways relevant to the nervous system [43]. Clinical studies on a number of neuroprotective anti-inflammatory drugs have been conducted for a range of neurological disorders and traumas; however, despite FDA approval, the majority of these trials have produced unsatisfactory results, since the drugs do not have a superior impact on cognitive function [44,45]. Drug repurposing such as monoclonal antibodies (mAbs) has been used, as in other situations, for the treatment of neurological illnesses, saving time and money. Reusable drugs include azithromycin, which was tested in a preclinical study and showed a reduction in ischemic brain injury (22 h) after transient (2 h) middle cerebral artery occlusion (MCAO) in adult male rats [46]. Another example is tetracycline, which inhibited the morphological activation of microglia in the area adjacent to the infarct and also inhibited the induction of IL-1β-converting enzyme and decreased the expression of cyclooxygenase-2 and the production of prostaglandin E2 [47,48]. These novel methods have been demonstrated to be successful in treating diseases that were previously incurable, particularly when the diseases have comparable biological properties. Since 2017, mAbs have been used to treat a number of neurological conditions, opening up new therapeutic possibilities: Ocrelizumab for multiple sclerosis; Eculizumab for neuromyelitis optica spectrum disorder (NMOSD) or myasthenia gravis; Fremanezumab, Erenumab and Eptinezumab for migraine; Galcanezumab for chronic migraine and cluster headache; and Satralizumab, Inebilizumab and tocilizumab for NMOSD (Figure 2 and Table 1). These humanized IgG monoclonal antibodies were authorized for treatment in various neurological diseases by the FDA 4 years ago [49,50,51,52,53,54] (Table 1). Ocrelizumab was approved for primary progressive and relapsing remitting multiple sclerosis by the FDA in 2017 [55] (Table 1). This fully humanized IgG CD20 antibody was the first drug approved for primary progressive multiple sclerosis. Administration by infusion provokes more frequent and intense local reactions compared with other monoclonal antibodies [56]. In a phase III double-blind, placebo-controlled trial, Ocrelizumab was found to be superior to placebo across a number of efficacy measures, including a reduction in the proportion of patients who experienced confirmed disability progression at 12 and 24 weeks and a reduction in the proportion of brain volume loss [57]. In patients with neuromyelitis optica spectrum disease (NMOSD), demyelinating inflammation begins in the optic nerves, spinal cord, and brainstem as a result of humoral response directed against astrocytes. Pathogenic anti-aquaporin-4 (AQP4) antibodies have been linked to NMOSD in the majority of patients [58]. To treat adults with neuromyelitis optica spectrum disorder (NMOSD) who are seropositive for immunoglobulin G autoantibodies against AQP4-IgG, the Food and Drug Administration (FDA) approved the humanized monoclonal antibody, Inebilizumab, in 2020 [59] (Table 1). Tocilizumab is an antibody that blocks the signaling of the interleukin 6 receptor (IL-6R) [60] (Table 1). Increased synthesis of interleukin 6 (IL-6) and its subsequent enhancement of AQP4-IgG secretion has been linked to NMOSD. Tocilizumab has been demonstrated to be beneficial for individuals with NMOSD who have not responded to conventional therapy [61,62,63]. The approval of Satralizumab as a disease-modifying medication for anti-AQP4 seropositive NMOSD was based on the results of two phase III studies [64,65], making it the second humanized anti-IL-6 receptor IgG2 mAb to achieve this distinction. Eculizumab, a humanized antibody, has also been shown to be effective in the treatment of NMOSD, with recurrence rates decreasing from 43% to 3% in the treated group in patients with AQP4-IgG-seropositive NMOSD [66] (Table 1). It is well established that autoantibodies targeting AQP4 mediate their cytotoxic action through the complementary stimulation [67,68]. The AQP4 is a glial membrane water channel, highly expressed in astrocytic processes adjacent to cerebral capillaries and membranes lining the subarachnoid space [69,70]. A study in mice deficient in AQP4 showed that AQP4 inhibition may provide a new therapeutic option for reducing brain edema in a wide variety of cerebral disorders [71], which suggests that AQP4 could be a potential target for cerebral ischemia. Currently, this target has not been tested for brain stroke. 

Eculizumab blocks the terminal complement protein (C5) stimulation pathway by interacting with C5 with strong affinity and specificity [72]. Interestingly, the complement protein-target could be a potential target to be tested for cerebral ischemia therapy. Clinical studies with Eculizumab, Inebilizumab, and Satralizumab showed that these mAbs significantly lowered both the yearly recurrence rate and disability (mean expanded disability status scale score drop −0.51, 95% CI: −0.92 to −0.11, *p* = 0.01). Subpopulation results revealed that Eculizumab was more successful in reducing the on-trial recurrence probability in anti-AQP-4+ individuals [73]. Eculizumab was approved by the FDA in 2018 and 2019 for NMOSD and MG, respectively (Table 1). The FDA has also recently authorized four monoclonal antibodies (mAbs) for use as preventative therapies for migraine. They are all aimed targeting a specific mediator in the disease’s etiology known as calcitonin gene-related peptide (CGRP). As of now, mAbs are the only disease-specific and mechanism-based prophylactic for both episodic and chronic migraine. Eptinezumab, Fremanezumab, and Galcanezumab target the CGRP ligand, whereas Erenumab targets the CGRP receptor [74,75,76,77,78,79,80] (Table 1). Erenumab is the only completely human mAb that targets the CGRP receptor. In a meta-analysis of phase III studies using anti-CGRP mAbs for both episodic and chronic migraine, 50.8% (95% CI 44.9–56.6%) of patients saw at least a 50% reduction in mean migraine-days per month. Galcanezumab has also shown efficacy in treating cluster headaches [81]. Low dropout rates in clinical trials attest to their great tolerance and attractive risk-benefit profile [82,83]. This has ushered in a new era in the preventative treatment of migraine. Although Fremanezumab, Galcanezumab, and Erenumab all showed good tolerance and sustained increases in numerous effectiveness metrics throughout the course of long-term open-label studies lasting more than a year [84,85,86], their expensive price tag is the biggest downside. CGRP is an excellent endogenous vasoactive substance reported to be involved in the attenuation of cerebral ischemia [87]. A study showed that CGRP significantly reduced post-ischemic increase of brain edema with a 2-h therapeutic window in the transient model of focal cerebral ischemia [88]. Moreover, it seems that at least part of the anti-edematous effects of CGRP is due to decrease of BBB disruption by improving ultrastructural damage of capillary endothelium cells, enhancing basal membrane, and inhibiting AQP4 and its mRNA over-expression [89]. The data of the present study provide a new possible approach for acute stroke therapy by administration of CGRP or inhibiting the CGRP molecule with mAbs (Figure 2 and Table 1).

This suggests that the CGRP molecule may be a potential mAb target for stroke. Similarly, the study showed that IL-6, produced locally by resident brain cells promotes angiogenesis in the delayed phases of stroke recovery [90]. Moreover, IL-6 is a cytokine originally identified as a B-cell differentiation factor nearly 30 years ago and is capable of inducing the maturation of B cells into antibody-producing cells [91]. As with many other cytokines, it has been recognized that IL-6 is not only a factor involved in immune response but has many critical roles in major physiological systems, including the nervous system [91]. Its expression is affected in several of the major brain diseases, including cerebral ischemia, and animal models strongly suggest that IL-6 may play a role in the observed neuropathology and is therefore a clear target for therapies in cerebral ischemia [92].

Through the reviewing of recent advances in successful antibody-based drugs for neurological disorders, we emphasized the potential in repurposing of these effective antibody drugs for the treatment of cerebral ischemia in the near future. As the increasing amount of evidence showing the pathological roles of such targets as CD19, IL-6, C5 complement, CGRP, and CD20 in the modulating of cerebral ischemia severity in clinical reports or pre-clinical studies [93,94,95,96,97], we believe these currently available antibody-based drugs for different neurological disorders could soon been evaluated and re-purposed as an effective antibody drug for stroke treatment in clinic.

## 4. Application of Antibody-Based Drugs for Cerebral Ischemia

The use of the antibodies in stroke therapy further extends the window for treatment from days to weeks or even months after the stroke and enables the treatment of chronic strokes months after the acute infarction. In addition, various contributory factors, such as pro-inflammatory cytokines, make stroke treatment unfeasible. Stroke outcomes may be improved if monoclonal antibodies (mAbs) are utilized to target the inflammatory events. mAbs can be used to block the damaging processes connected to inflammation by blocking pro-inflammatory cytokines and their receptors. For instance, brain damage following an acute stroke is partially caused by ion channels and neurotransmitter receptors [98]. One potential therapy to treat the effects of acute stroke is the development of specialized monoclonal antibodies (mAbs) such as nanobodies that target particular inflammatory signaling pathways, cell death pathways, ion channels, and neurotransmitter receptors [98]. Nanobodies have the following special properties: small size of less than 400 daltons, stability in aqueous solutions, reversible folding, specificity, and high affinity for a single molecular target in the brain. These advantageous characteristics have compelled a number of academic research organizations, pharmaceutical companies, and biotech firms to utilize nanobodies as research tools or to develop new diagnostic and therapeutic applications.

Recent articles demonstrated the use of nanobodies in different animal models including cerebral stroke [99,100,101]. Experimentally, studies showed that nanobodies can penetrate epithelia or the blood–brain barrier [102,103,104,105]. Similarly, numerous studies have demonstrated that toll-like receptors (TLRs) are a key mediator of ischemic brain damage [106,107]. Following cerebral ischemia, TLR4 is strongly activated [108]. TLR4 defective mice were shown to be protective against ischemic stroke in several investigations [107,108,109,110]. Following the establishment of localized cerebral ischemia, intravascularly administered monoclonal antibodies penetrate the rodent brain [111]. The anti-inflammatory impact of TLR4-inhibition in experimental stroke was specifically established by the administration of TLR4 blocking antibody (mAbs clone MTS510) in vivo [112] (Table 2). In light of these findings, it is clear that intravascular TLR4 antibody administration effectively guards against ischemia damage for at least 48 h following reperfusion. These facts suggest that treating cerebral ischemia using antibody-based medications might result in a cure.

**Figure 3:**
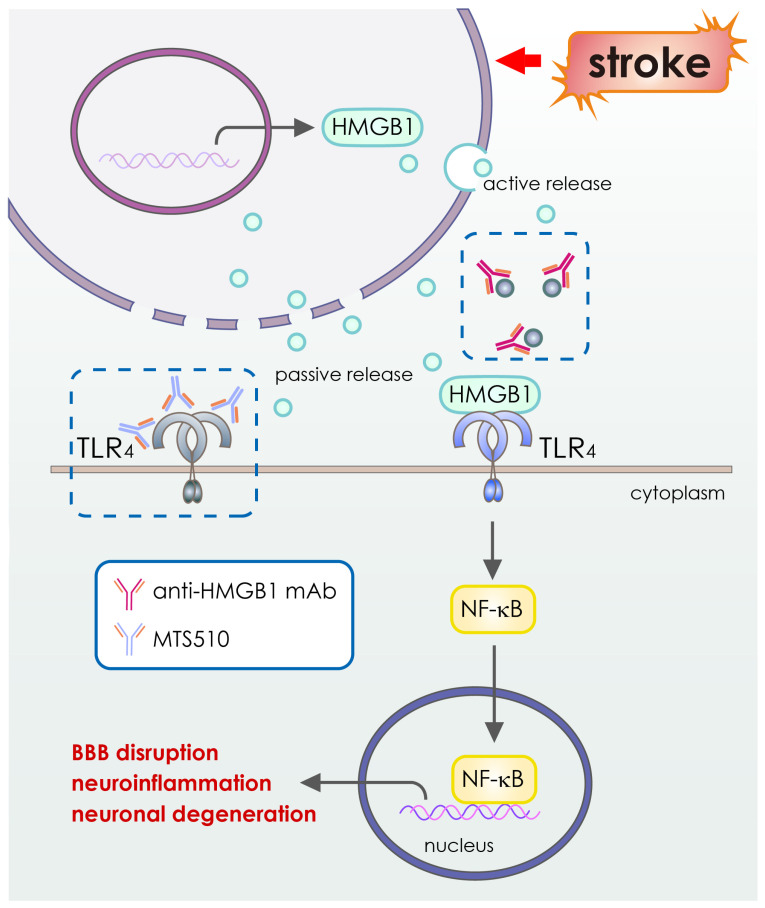
Schematic illustration of function of HMGB1 or TLR4 in the pathology of stroke. The high mobility group box 1 (HMGB1) protein is a typical damage-associated molecular pattern (DAMP) protein that can bind to the toll-like receptor 4 (TLR4) and initiates and activates the NF-κB signaling axis. The activation of the NF-κB pathway significantly increases the production of pro-inflammatory cytokines, including TNF-α, IL-6, and IL-1β, which causes exaggerated neuroinflammation, BBB disruption, and neuronal degeneration after cerebral ischemia. Inhibition of TLR4 or HMGB1 with antibody-based drugs can ameliorate the neuroinflammatory responses and neuronal damage after stroke.

**Figure 4:**
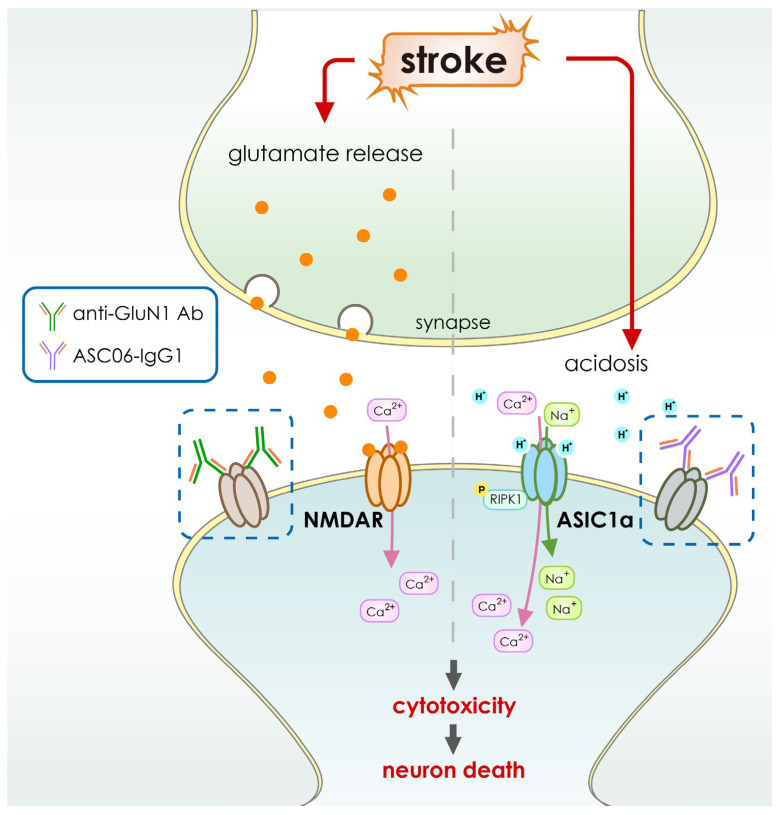
Schematic illustration for the function of ASIC1a or NMDAR in the development of stroke. Acidosis caused by anaerobic glycolysis is a common side effect of stroke. When pH levels drop after stroke, ASIC1a is activated, allowing calcium to flood the cell, creating an improper ionic balance and osmotic pressure. On the other hand, glutamate is the chief excitatory neurotransmitter in the central nervous system and displays critical roles in synaptic communications and flexible synaptic plasticity. However, the extensive and rapid accumulation of glutamate at the synaptic cleft after ischemic stroke can lead to over-excitation of the NMDARs (N-methyl-D-aspartate receptors), which can eventually be lethal to neurons. Both of the membrane-originated pathological events lead to edema and depolarization, which in neurons causes massive excitotoxicity from increased intracellular calcium ions and eventually, inflammation, hence ischemia injury. Inhibition of the ASIC1a or GluN1 with antibody-based drugs can reverse the neuronal death and protect against cerebral ischemia.

In cerebral ischemia, tissue acidosis is a known factor in neuronal cell death. Therefore, better treatment options for neuroprotection can be created with a deeper knowledge of the signaling mechanisms underlying acidotoxicity. Acid-sensing ion channels (ASICs), and more specifically, subtype ASIC1a, play a crucial role in acidosis-induced damage to brain neurons among the different proton-sensing systems [119,120]. The protective impact of ASIC1a gene deletion and/or pharmacological blockage in animal models of ischemic stroke [119] demonstrates the important involvement of ASIC1a in neuronal acidotoxicity. During inflammatory and ischemic brain injuries, local tissue acidosis and the activation of ASICs occur [121,122] (Figure 3). The ASIC1a subtype was shown to be the primary ASIC protein activated in neuronal damage [121,123]. This subtype seems to be permeable to both sodium and calcium and has a high sensitivity to protons [124]. Due to a lack of oxygen, anaerobic metabolism develops and lactic acid accumulates outside the cells in patients suffering from a severe ischemic stroke. Neuronal injury is triggered by extracellular acidosis, a temporary rise in calcium generated by the opening of the channel [121]. When activated, ASIC1a has a greater proton permeability than sodium [119], suggesting that acidosis-induced cell death may be caused by an inflow of protons. Thus, ASIC1a activation, which allows calcium ions through, is a therapeutic target in neuronal damage [125,126]. A highly specific and effective antibody against ASIC1a was therefore developed. When tested on a mouse model of stroke, researchers found that infusing the animals with this antibody decreased brain ischemic damage [121,123,127,128] (Figure 4). Because of this, these studies show that targeting ASIC1a with this antibody-based medicine could be used in the future to treat cerebral ischemia.

**Figure 5:**
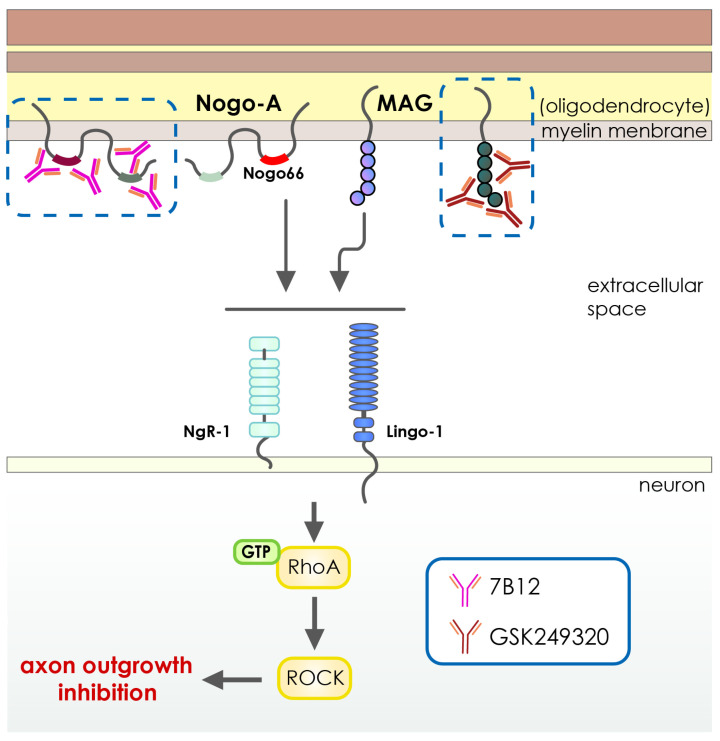
Schematic illustration of MAG or Nogo-A signaling cascades in the pathology of cerebral ischemia. The overproduction of Myelin-associated glycoprotein (MAG) Neurite outgrowth inhibition protein-A (Nogo-A) after cerebral ischemia activates the signaling cascades linked to the RhoA/ROCK pathway, which collapses neuronal growth cones and inhibits axonal growth. Such inhibitory events significantly suppress the functional recovery after the suffering of cerebral ischemia. Inhibition of the MAG or Nogo-A with highly specific antibody-based therapeutics can enhance the axonal regeneration and neurite outgrowth dramatically, which leads to better functional recovery after cerebral ischemia.

Meanwhile, some other antibody-based therapies have also been studied for their potential to treat stroke. Examples include anti-GluN1 antibodies, which block N-methyl-D-aspartate (NMDA) receptors linked to calcium influx [114] (Table 2 and Figure 4), anti-High Mobility Group Box-1 antibodies, which target High Mobility Group Box-1, an inflammatory protein [115] (Table 2 and Figure 3), the GSK249320 antibody, which is aimed at myelin-associated glycoprotein (MAG) [116], and the anti-Nogo-A antibody, 7B12, which targets the Nogo A myelin (NAM) associated neurite outgrowth protein [117]. Antibodies (MAb) enhance neuronal healing by binding to receptors and surface markers and blocking or neutralizing growth-inhibiting factors in neurons [98]. Myelin-associated glycoprotein (MAG), oligo-myelin glycoprotein, and Nogo-A are three primary inhibitors of neurite outgrowth and neuronal regeneration. MAG, oligo-myelin glycoprotein, and Nogo-A are increased [129,130] after an acute ischemic stroke (Table 2 and Figure 5). The inhibition of neurite outgrowth and neuronal regeneration by the MAG or Nogo-A signaling cascades block the functional recovery after the suffering of cerebral ischemia. Neuronal healing and axonal rewiring are boosted after an acute ischemic stroke when these inhibitors are blocked by MAb [98] (Figure 3 and Table 2). Because of this, monoclonal antibodies (mAbs) are being considered as possible therapeutic agents for stroke patients [131,132]. Animal models of middle cerebral artery occlusion (MCAO) show that the vast majority of mAbs used to treat stroke are effective at reducing infarct volume and improving neuronal function [98].

Furthermore, cerebral ischemia induces sterile inflammation that exacerbates initial brain injury and neurologic outcome [133,134]. Inducing molecules include adenosine triphosphate (ATP) as well as nicotinamide adenine dinucleotide (NAD), heat shock protein (HSP), and high mobility group box 1 protein (HMGB1). These molecules activate various signaling pathways, such as the ATP/P2X7 signaling pathway or the Nuclear Factor Kappa-Light-Chain-Enhancer of Activated B Cells (NFκB) signaling pathway [135,136]. One study shows that there is a substantial local increase in ATP after cerebral ischemia and that targeting the ATP receptor P2X7 with P2X7-specific antibodies could reduce ischemic tissue damage [118]. 

Meanwhile, a recent preclinical, randomized, controlled, multicenter trial testing cerebral ischemia therapy in a mouse model focused on anti-CD47d antibodies that inhibit leukocyte migration into the brain [137]. The study found that treatment with CD49d-specific antibodies significantly reduced both leukocyte invasion and infarct volume after permanent distal middle cerebral artery occlusion caused a small cortical infarct [137]. In contrast, anti-CD49d treatment did not reduce lesion size or affect leukocyte invasion after transient proximal middle cerebral artery occlusion that resulted in large lesions [137]. These results suggest that the utility of immunologic approaches may depend on the severity and location of the infarct.

In addition to this recent progress in antibody-based drugs for cerebral ischemia, as mentioned previously, the antibody-based drugs currently available for different neurological disorders (Table 1 and Figure 2) have great potential to be repurposed as an effective antibody drug for stroke treatment in the near future.

## 5. Pros and Cons of Antibody-Based Drugs for Cerebral Ischemia

A major obstacle to the current application of mAbs is the lack of similarities between humans and the preclinical models used for testing. The inflammatory responses, expression of receptors, and prevalence of targets elicited in humans and rats/mice may differ, limiting the success of mAbs in the clinic. Dose optimization for mAbs requires careful evaluation to eliminate the risk of adverse side effects such as bleeding during clinical trials [37]. Clinical research addressing the efficacy and safety of mAbs must be conducted to maximize the therapeutic benefits of mAbs. The use of nanobodies, i.e., antibodies isolated from alpaca, camel, llama, or shark heavy chain antibodies, may be more beneficial in cerebral ischemia because they improve BBB and tissue penetration and lend themselves to surface modification to allow site-specific targeting [38]. The following table provides an overview of both the benefits and the challenges associated with using antibodies as a treatment for cerebral ischemia (Table 3). 

There are two possible explanations for the lack of success: (i) inadequate uptake of the antibody into the CNS via the BBB, and (ii) administration of the antibody at a stage in the course of the disease that is too late [138]. In this sense, the absence of biomarkers that can detect the early stages of disease is one of the most significant challenges that contemporary medicine needs to address and overcome. In spite of this, the most important step in the process of developing antibodies to treat disorders of the brain is to determine whether or not antibodies that are given in the periphery can pass the BBB, and if so, in sufficient quantities to trigger a therapeutic response in the brain itself. However, some of the failures of these mAbs that have been recorded were also related to safety problems, including both cerebral microhemorrhages and meningoencephalitis. It is possible that this is caused by an accumulation of mAb in the brain as a result of a low level of reverse transfer from the brain to the blood. Therefore, understanding antibody influx and efflux from the blood to the brain and back is going to be the most important challenge for pharmaceutical companies and academic groups to tackle in the near future. This will allow them to develop therapeutic antibodies that are safer and more effective for diseases that affect the central nervous system.

## 6. Strategies to Improve the Efficacy of Antibody-Based Drugs for Cerebral Ischemia

**Figure 6:**
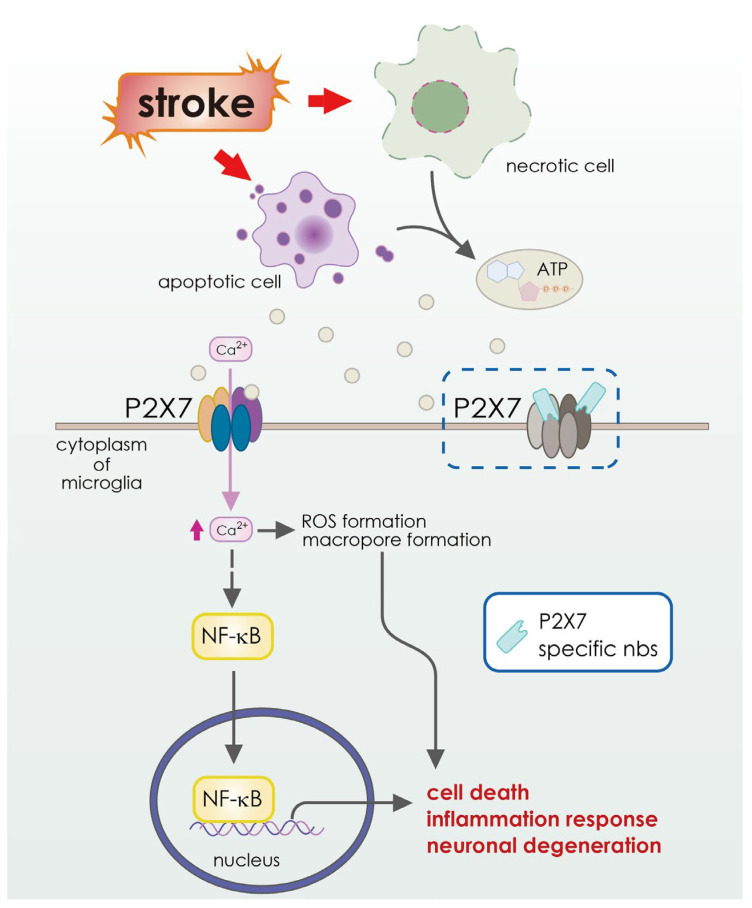
Schematic illustration of ATP and P2X7 signaling cascades in the pathology of cerebral ischemia. Under cerebral ischemia, energy deprivation causes anoxic and irreversible depolarization, which leads to excessive release of glutamate and ATP. ATP could act as a damage-associated molecular pattern (DAMP) that activates P2X7 receptors on both neurons and glia cells. Neuronal-expressed P2X7 receptors initiate calcium overload and extensive excitotoxic neurological injury. On the other hand, microglia-expressed P2X7 receptors regulate its aberrant activation and over-production of reactive oxygen species and pro-inflammatory cytokines. These ATP-P2X7 signaling cascades modulate immune responses, ROS production, and eventually progressive neurological damage.

The development of chimeric, humanized, or fully human antibodies was a major breakthrough and led to a wave of antibodies approved by the FDA. Currently, more than 22 antibodies are marketed as therapeutics, mainly for the treatment of cancer and immune disorders. Impressive results have been achieved in cancer therapy, as demonstrated by the success of rituximab in the treatment of various cancers. However, this is not true for the use of antibodies to treat strokes due to the blood–brain barrier (BBB). The antibodies cannot cross this barrier. However, the use of cell receptor-mediated strategies would improve the efficacy of antibodies in treating strokes. This natural strategy takes advantage of the various receptors expressed at the BBB, as well as the physiological properties of the BBB (e.g., charge and lipid composition) [139,140]. These translocation mechanisms are fundamental for the uptake of essential substances to maintain brain homeostasis. They fall into several categories depending on their specific roles, including receptor-mediated transport (RMT), which is responsible for the translocation of large molecules such as transferrin or antibodies (Figure 6). To date, no study has exhausted the potential of the receptors. This receptor-mediated strategy achieves the goal of reaching the site of the brain of stroke patients in order to efficiently overcome the brain barrier [141]. These receptor-targeted molecules can be either peptides or antibodies [142]. 

Accumulating evidence suggests that certain BBB enriched receptors, such as transferrin receptor (TfR), insulin receptor (IR) and Diphtheria toxin receptor (DTR), low-density lipoprotein-related protein (LDLRP), insulin-like growth factor receptor (IGFR), nicotinic acetylcholine receptor (NACR), leptin receptor (LEPR) and Scavenger receptor, Class B, and Fc fragment of IgG receptor transports alpha (FCGRT) could be potentially conjugated to increase the efficacy of receptor-mediated transport of antibodies into the brain (Table 4). TfR and IR are the main BBB receptors that have been extensively studied by researchers. Pardridge et al. comprehensively documented the use of antibodies targeting these receptors [143,144,145]. In vivo studies showed accumulation of various anti-TfR monoclonal antibodies (mAbs) in the meninges and clear biodistribution. However, the high affinity of the antibodies for these receptors is a limitation because it leads to weak dissociation of the receptors. Consequently, the high-affinity antibodies follow the lysosome pathway during intracellular trafficking, which leads to their degradation [146]. Yu et al. elegantly solved the problem by reducing the affinity of anti-TfR antibodies to the receptors [147]. Similar to TfR, there are exciting studies targeting IR. Pardridge et al. demonstrated an overall 4% uptake in the brain 3 h after intravenous administration in rhesus monkeys. More importantly, effective targeting primarily requires an understanding of the physiology and anatomy of the blood–brain barrier. The other approach is to modify the surface of the drug-loaded nanoparticle by conjugating it with an antibody that is particularly specific to the brain, such as the OX26 antibody and the transferrin receptor. The antibody-drug conjugate recognizes a specific ligand (in this case, the transferrin receptor) and crosses the blood–brain barrier by receptor-mediated endocytosis. Antibodies are the most promising tools for targeting the blood–brain barrier receptors because of their ability to bind specifically. For example, antibodies targeting transferrin and insulin receptors can act as transporters to cross the blood–brain barrier and deposit drug molecules via receptor-mediated endocytosis. Several other therapeutics, like nerve growth factor and brain-derived neurotrophic factor, are also transported across the blood–brain barrier upon binding with the transferrin receptor antibody [148]. Another instance is the significantly increased uptake upon conjugation with a monoclonal antibody against the human insulin receptor following intravenous administration [149]. The table shows the existing endogenous brain barrier transporter strategies, such as receptor-mediated transport (RMT) [150].

RMT expressed on the endothelial surface of the BBB can be targeted by the genetically modified molecules (Figure 6). Other transporters include carrier-mediated targets (CMT) and (AET), which are in charge of transporting small molecules between the blood and brain such as glucose, nucleosides, and amino acids but are ineffective for moving big molecules [161]. Adsorptive-mediated target (AMT) is a non-specific process that transports a number of cationized proteins into the brain using a similar mechanism to a receptor-mediated process. It starts when cationic serum proteins interact with negatively charged areas on the endothelial luminal surface. Thereafter, proteins are delivered to the abluminal surface of endothelial cells and released into the brain by exocytosis [162,163].

**Figure 7:**
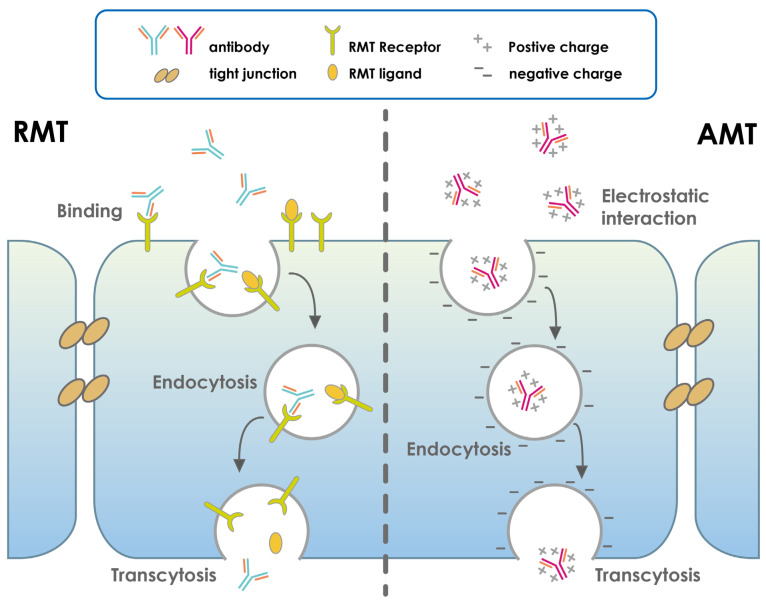
Sketch illustration of the brain’s processes for antibody-based drug transport via the blood–brain barrier. In a receptor-mediated target (RMT), the ligand and antibody in the blood specifically bind to RMT receptors in the apical cell membrane; (2) the receptors and ligands are internalized in an energy-dependent manner to form an intracellular vesicle; and (3) these vesicles are shuttled through the cell cytoplasm, and the antibodies and ligands are released into the brain via the fusion of vesicles with the basolateral cell membrane. In an adsorptive-mediated target (AMT), the positively charged antibodies interact electrostatically with the negatively charged apical cell membrane, forming intracellular vesicles containing antibodies through energy-independent endocytosis. Finally, after traveling through the cell cytoplasm and fusing with the basolateral cell membrane, these vesicles release their contents into the brain.

In contrast, RMT systems are responsible for the transport of certain endogenous large molecules across the BBB (Figure 6). In RMT, molecules in the bloodstream can bind to specific receptors on the luminal surface of the brain. After binding, the receptor-ligand complex is internalized into the endothelial cell by receptor-mediated endocytosis. The ligand molecule is then transported into the brain through the abluminal membrane of the endothelial cell [141]. Many studies have targeted the RMT for its properties to develop molecules that can efficiently cross the BBB and deliver a therapeutic CNS drug to the brain, such as monoclonal antibodies that specifically target RMT receptors [164,165,166,167]. mAbs have long been an important tool in basic research due to their high specificity and affinity for target antigens. In addition, mAbs have significantly impacted medical care in a variety of diseases over the past two decades, as evidenced by the growing number of therapeutic mAbs on the market and in clinical trials, as well as the development of new recombinant antibody constructs as therapeutics [168]. For these reasons, recombinant antibodies against RMT receptors have a promising potential to be used as delivery vehicles to the brain for the treatment of cerebral ischemia (Figure 7).

**Figure 8:**
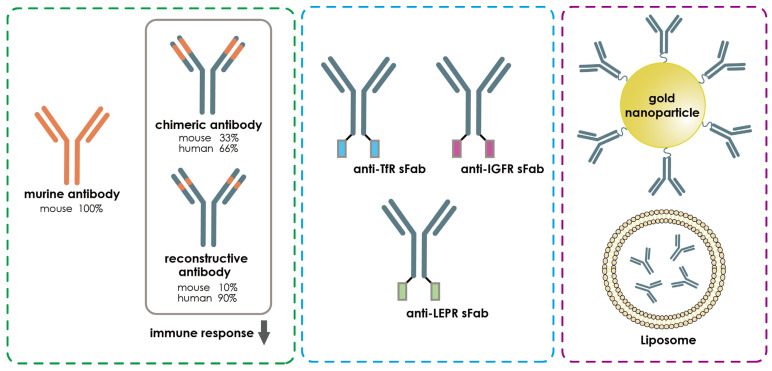
Strategies to improve the efficacy of antibody-based drugs for cerebral ischemia. The over-activation of immunological responses by the antibody drugs could cause serious biological and clinical consequences. The recent development of fully human or humanized antibodies has greatly reduced the risk for inducing exaggerated immune responses compared to the mouse or chimeric antibodies. Meanwhile, bivalent conjugation of an antibody of BBB enriched receptors, such as TfR, LDLRP, or NACR, with the target antibody drug could significantly increase the efficacy of receptor-mediated transport of antibodies into the brain. Nanoparticles or liposomes, which prevent the degradation of antibody drugs and show better efficacy in BBB penetration, can be used as vehicles to transport the antibody into the brain after cerebral ischemia.

Furthermore, nanoparticles can be used as a vehicle to transport the antibodies across the BBB [169]. They have shown many advantages. For example, they can pass through the BBB because of their small sizes, often are between 1 and 100 nanometers [169]. Additionally, they have fewer side effects due to their high specificity. Nowadays, different nanoparticles such as gold and liposomes can be combined with antibody fragments including scFv, Fab fragment, and single domain antibody fragment to treat cerebral ischemia. Their combination could facilitate the penetration of the BBB and provide multiple therapeutic effects [170]. Gold nanoparticles (AuNPs) have excellent biocompatibility and stability properties [170]. Their uses make them outstanding candidates in comparison with other nanoparticles (Figure 8). Like gold nanoparticles, liposomes are another excellent example in the field of nanoparticles. They have been widely used as carriers (Figure 8). Therefore, they can be applied in the combination with antibody drugs for cerebral ischemia therapy. Liposome contains an aqueous core and a lipid bilayer, whose unique structure is able to carry many different kinds of antibody-based drugs through the blood–brain barrier [171,172].

## 7. Clinical Translation of Antibody-Based Drugs for Cerebral Ischemia

Despite the fact that clinical trials of antibody-based drugs are currently underway, there are a number of obstacles that prevent these approaches from being successful in providing clinically relevant therapeutic interventions for cerebral ischemia. One of these is the BBB, whose role is to prevent chemicals from entering the brain by preventing them from crossing the bloodstream [173]. Stroke pathology leads to the activation of a number of signaling pathways, each of which acts as a preliminary obstacle to achieving the required level of efficacy [174]. However, this obstacle can be overcome by developing carriers with the ability of multimodal targeting. On the other hand, research in animal models of stroke has shown that certain inflammatory processes can positively influence the recovery process after stroke [99,100]. These findings were published in the Journal Stroke. According to the findings of a phase I/II study [116,175], the monoclonal antibody drug SAN DIEGO -GSK249320 appears to be safe for patients who have previously suffered a stroke. By encouraging axonal growth and counteracting the inhibition induced by myelin-associated glycoprotein (MAG), this medication has the potential to enhance the outcomes for patients who have suffered a stroke [47]. It is possible that SAN DIEGO -GSK249320 reverses the inhibition caused by MAG. In addition, there is no evidence that a single infusion of natalizumab increases the risk of infection or other serious adverse effects in people with multiple sclerosis [85], and these patients report that they tolerate the drug well. In the study ACTION, a phase 2 trial, patients who had recently suffered an acute ischemic stroke were given an intravenous infusion of natalizumab. This allowed researchers to assess both the safety and efficacy of the treatment for these patients. Currently, there are not many different treatment options [176]. This is despite the fact that many antibody-based treatment platforms for cerebral ischemia are still in development. However, there is a lack of appropriate animal models to test the safety and efficacy of antibody-based drugs in the treatment of cerebral ischemia [177,178]. This makes it difficult to determine whether these drugs are effective. It is necessary to find a solution to this problem. 

Another factor that contributed to the difficulties was the high mortality rate of the animals during the study. We need a robust platform on which to test models of brain stroke if we are to successfully overcome all these challenges [179]. This platform should be useful to evaluate neuroprotective benefits, and it should also increase the success rate of antibody-based therapeutic approaches when tested in clinical trials [180]. The development of second-generation recombinant antibodies has made it possible to use more than 20 different mAbs for therapeutic purposes, resulting in a significant collection of clinical data. Researchers and antibody developers are currently using the clinical results to direct their efforts toward new methods that will lead to even more effective antibodies. Initial clinical trials are currently underway to test a new class of antibody drugs. These drugs have the potential to be much more successful than conventional mAbs, with the possibility of developing immunotherapeutic drugs that are even more effective in the future. This shows that antibody engineering will indeed have a significant positive impact on health, as expected.

Another study showed that delayed treatment with tissue plasminogen activator (tPA) increased the risk of intracerebral hemorrhage in patients with cerebral ischemia [181]. A study also showed that treatment with tPA caused hemorrhagic complications in a 4-h mouse model of middle cerebral artery occlusion (MCAO) when administered after reperfusion. In the same 4-h MCAO mouse model, anti-High Mobility Group Box 1 (αHMGB1) antibody therapy was tested to evaluate the benefit of αHMGB1 antibody treatment in the delayed phase of ischemia, outside the therapeutic time window of tPA [181]. The results showed that αHMGB1 antibody treatment reduced infarct volume and swelling and improved neurological impairment and motor coordination without hemorrhagic complications by inhibiting HMGB1 activity. In addition, the αHMGB1 antibody suppressed pathways of secondary inflammatory responses, such as IL-6 and TNF-α, after cerebral ischemia [181]. This study suggests that treatment with an αHMGB1 antibody may be an effective therapeutic option for patients who exceed the therapeutic window for tPA. Another randomized, controlled, open-label, phase II clinical trial with blinded outcome assessment was conducted [182]. This phase II study targeted one of the inflammatory molecules (complement C5 factor protein). The inflammatory response after aneurysmal subarachnoid hemorrhage (aSAH) has been associated with early brain injury, delayed cerebral ischemia, and poor functional outcome [182]. In an experimental study SAH, administration of C5 complement antibody shortly after SAH reduced brain injury by approximately 40% [182]. This study evaluated the pharmacodynamic efficacy and safety of eculizumab (a C5 complement antibody) in patients with aSAH. Eculizumab (1200 mg) was administered intravenously at < 12 h, day 3, and day 7 after ictus (a stroke, specifically a cerebrovascular accident) [182]. Patients in the intervention group received prophylactic antibiotics for 4 weeks and prophylactic antifungal therapy if the patient had central access or an external ventricular shunt and a positive fungal or yeast culture [182]. The trial is ongoing (not completed) but has successfully passed phase I (Netherlands Trial Register: NTR6752).

## 8. Limitation of Antibody-Based Drug for Cerebral Ischemia

The major challenges are that the complete antibody cannot cross the BBB unless one arm of the binding site is targeted to the receptor on the endothelial cells of the BBB. Another limitation is immunogenicity. If the antibody is antigenic, it will be removed from the parenchyma by the brain’s immune system. A further limitation is the fear of lack of efficacy in clinical trials. Most antibodies are studied in animal models, and it is not clear whether they work in humans as they do in animal studies.

## 9. Conclusions and Future Direction

The production of antibodies for the treatment of stroke still has a long way to go before it can be considered successful. Although a number of strategies, such as traditional antibodies, have been investigated, new techniques are now being developed. The results obtained so far with traditional antibodies have not been convincing, and there is not yet enough evidence to determine their exact mode of action. Nevertheless, much remains to be done to successfully deliver therapeutic doses of these mAbs to the brain and to ensure that they are safely cleared from the brain. Recent evidence that delivery of therapeutic antibodies to the CNS can be greatly improved by linking a therapeutic antibody to an antibody that crosses the BBB has fueled hope that therapeutic antibodies may one day be delivered directly to the nervous system. The techniques described above clearly demonstrate that there are many new methods to modify antibodies to better penetrate the BBB. These methods offer tremendous advantages by increasing the delivery of the injected dose and reducing the amount of antibody required for injection, which in turn reduces drug exposure and improves safety.

Despite these difficulties, efforts are currently concentrated on creating additional types of antibody therapies that can penetrate or somehow interact with the BBB for the best in vivo results. For the time being, brain targeting may not be possible using TfR receptors to deliver antibodies across the BBB. However, studies using the anti-TfR antibodies that are currently available have shed light on the BBB receptors’ modes of action and highlighted challenges in protein engineering that need to be addressed before an effective BBB shuttle can be created. The lack of observed species cross-reactivity in the existing antibodies is another difficulty with bringing anti-TfR antibodies to the clinic. To address this issue, either transgenic mice must be created to produce human antigens that tolerantly accept humanized antibodies, which will add significantly to development expenditures, or antibodies should be created for usage in each the species under inquiry. Additionally, all of the previously mentioned parameters, including target interaction, FcRn binding, molecular size, and surface charge, are likely to contribute to the movement of anti-receptor antibodies across the BBB in the search for a way to increase the penetration of antibodies in the brain for the successful treatment of cerebral ischemia.

## Figures and Tables

**Table 1:** Antibody-based drugs approved during 2017–2021 for neurological disorders.

Generic Names	Antigens	Designs	Neurological Conditions	Administration	FDA Approval	Funders
Inebilizumab-cdon	CD19	Humanized IgG1	Neuromyelitis optica and neuromyelitis optica spectrum disorders	Intravenous infusion	2021	Viela Bio
Satralizumab-mwge	IL-6R	Humanized IgG2	Neuromyelitis optica spectrum disorders	Subcutaneous injection	2020	Genentech Inc.
Tocilizumab	IL-6R	Humanized IgG1	Neuromyelitis optica spectrum disorder	Intravenous infusion	2019	Chugai/Roche
Eculizumab	C5 complement protein	Humanized IgG2	Neuromyelitis optica spectrum disorder, Myasthenia gravis	Intravenous infusion	2019, 2018, respectively	Alexion Pharmaceuticals
Eptinezumab	CGRP ligand	Humanized IgG1	Episodic or chronic migraine	Intravenous infusion	2020	Lundbeck Seattle Biopharmaceuticals, Inc.
Galcanezumab-gnlm	CGRP ligand	Humanized IgG4	Cluster headache or chronic migraine	Subcutaneous injection	2019	Eli Lilly and Company
Fremanezumabvfrm	CGRP ligand	Humanized IgG2	Episodic or chronic migraine	Subcutaneous injection	2018	Teva Pharmaceuticals
Erenumab-aooe	CGRP receptor	Human IgG2	Episodic or chronic migraine	Subcutaneous injection	2018	Amgen and Novartis
Ocrelizumab	CD20	Humanized IgG1	Relapsing-remitting Multiple sclerosis	Intravenous infusion	2017	Hoffmann-La Roche

CGRP: calcitonin gene-related peptide; IL-6R: interleukin 6 receptor; CD: cluster of differentiation; IgG: immunoglobulin gamma; C5: complement factor 5.

**Table 2:** Evaluation of mAbs in animal models of cerebral ischemia.

Antibody	Antigen	Design	Route	Main Findings	Citation
mABs clone MTS510	TLR4	Recombinant	Intravenous infusion	Decreased infarct size, brain edema, and improved neurological performance.	[112]
ASC06-IgG1	ASIC blocker	Recombinant IgG1	Intravenous infusion	The infarct volume was 23% smaller when calcium transport was blocked compared to control group.	[113]
Anti-GluN1 antibodies	Blocks NMDA receptor associated calcium influx	Recombinant	Subcutaneous injection	Protects neurons from damage by preventing thrombus development and decreasing platelet aggregation.	[114]
Anti-High Mobility Group Box-1 mAbs	HMGB1, inflammatory proteins	Recombinant	Intravenous infusion	Brain–blood barrier (BBB) disruption, less oxidative stress, more powerful antioxidants, and less brain swelling.	[115]
GSK249320	MAG	Humanized recombinant IgG1	Intravenous infusion	Increases neurological function and penetrates the region of the lesion.	[116]
Anti-Nogo-A antibody 7B12	NAM-associated neurite outgrowth protein	Recombinant IgG1	Intravenous infusion	Improves sensory and behavioral outcomes and returns levels to pre-lesion levels, indicating effective penetration at the lesion site.	[117]
Anti-P2X7 antibodies	Adenosine triphosphate (ATP)	Recombinant nanobodies	Intravenous and icv injections	Reduced stroke size. Inhibits ATP-triggered calcium influx in microglial cells.	[118]

mABs: monoclonal antibodies; MAG: myelin-associated glycoprotein; ASIC: acid-sensing ion channel; TLR4: toll-like receptor 4; HMGB1: high mobility group box-1; NMDAR: N-methyl-D-aspartate receptor; NAM: nogo A myelin; P2X7: P2X purinoceptor 7.

**Table 3:** Advantages and disadvantages of antibody-based drugs in cerebral ischemia.

	Pros	Cons
1	Monoclonal antibodies are extremely sensitive and specific for antigens.	Injection-related reactions (IRRs). They occur most frequently in a period of 10 min to 4 h after the start of antibody administration. However, anaphylactic reactions are not expected to occur during the first mAbs infusion, except in the rare case that pre-existing immunoglobulins E (IgEs) cross-react with the infused mAb.
2	Without alteration, nanobodies such as VHH can cross the blood–brain barrier.	Cytokine release syndrome (CRS) depends largely on the cell type targeted by the mAb rather than its allergenic properties. mAbs that activate T cells are most likely to cause CRS, when large amounts of proinflammatory cytokines are released by activated astrocytes and white blood cells, including B cells, T cells, natural killer cells, macrophages, dendritic cells, and monocytes.
3	Antibodies are stable, especially nanobodies in extreme conditions such as temperature and pH.	The more immunogenic the mAbs, the more likely they are to produce anti-drug antibodies (ADA). This explains why ADAs are more likely to be produced against chimeric than human mAbs, and ADA production can lead to neutralization of mAbs, rapid elimination, and loss of efficacy.
4	Nanobodies can penetrate hard-to-reach epitopes.	Opportunistic infections occur when mAbs affect immune function by depleting cell populations (e.g., ocrelizumab) or blocking immune cell migration through endothelial barriers; dizziness.
5	Bispecific antibodies (BsAbs) can facilitate transport across the BBB. They are designed to contain one arm with specificity against a BBB RMT receptor that drives their migration across the BBB and a second arm with a therapeutic function that produces the pharmacological effect when the BsAb encounters the target.	MAb-associated malignancy. In a phase III study of the treatment of primary progressive multiple sclerosis with ocrelizumab, an anti-CD20, B-cell-depleting mAb, 11 cases of malignancy were reported in the active treatment group, 4 of which were adenocarcinomas of the breast [57]; back pain; diarrhea.
6	Antibodies can be easily cleared from the system without causing damage to the tissues as compared to chemotherapy.	Autoimmune disorders; nasopharyngitis

**Table 4:** The main blood–brain barrier transporters.

	Receptor-Mediated Targets (RMT)	Citations
1	Transferrin receptor (TfR)	[151,152,153]
2	Insulin receptor (IR) and Diphtheria toxin receptor (DTR)	[154,155]
3	Low-density lipoprotein-related protein (LDLRP)	[154,156]
4	Insulin-like growth factor receptor (IGFR)	[154]
5	Nicotinic acetylcholine receptor (NACR)	[154,155,157]
6	Leptin receptor (LEPR) and Scavenger receptor, Class B	[154,155,158,159]
7	Fc fragment of IgG receptor transports alpha (FCGRT)	[155,158,160]

## Data Availability

Not Applicable.
